# Serological evidence of exposure to *Bartonella* sp. in dogs with suspected vector-borne diseases, toxoplasmosis and neosporosis

**DOI:** 10.1590/S1984-29612022050

**Published:** 2022-09-19

**Authors:** Luiz Ricardo Gonçalves, Márcia Mariza Gomes Jusi Merino, Carla Roberta Freschi, Simone de Jesus Fernandes, Marcos Rogério André, Rosangela Zacarias Machado

**Affiliations:** 1 Imunodot Diagnósticos Veterinários – IMUNODOT, Jaboticabal, SP, Brasil; 2 Departamento de Patologia, Reprodução e Saúde Única, Faculdade de Ciências Agrárias e Veterinárias – FCAV, Universidade Estadual Paulista – UNESP, Jaboticabal, SP, Brasil

**Keywords:** Brazil, canine, seroreactivity, zoonoses, Brasil, canino, soro reatividade, zoonoses

## Abstract

Bartonellosis is a vector-borne zoonotic disease with worldwide distribution that infect a broad spectrum of mammalian species. Despite the recent studies carried out in Brazil, information regarding *Bartonella* in dogs are scarce. Therefore, we performed a retrospective study to investigate the exposure to *Bartonella* sp. in dogs by indirect immunofluorescence assay (IFA). Three hundred and thirty-five archived serum samples from dogs previously tested for vector-borne pathogens, *Toxoplasma gondii*, and *Neospora caninum* were screened for the presence of IgG antibodies to *Bartonella* sp. All dogs originated from the Metropolitan region of Ribeirão Preto, northeast of the State of São Paulo. Twenty-eight samples (8.3%) were positive for *Bartonella* sp. at the cut-off of 64. Among the 28 seropositive samples for *Bartonella* sp., 16 (57.1%) were also seropositive for *Ehrlichia canis*, 12 (42.8%) for *Babesia vogeli*, five (17.8%) for *T. gondii* and three (10.7%) for *L. infantum* and *N. caninum*. Our results demonstrated that dogs sampled were exposed to *Bartonella* sp. Since all the animals sampled in the present study were from private owners, our findings demonstrate that these people may also be exposed to *Bartonella* sp. Further studies designed to assess whether the infection by other arthropod-borne pathogens such as *B. vogeli* and *E. canis* are risk factors for *Bartonella* infection are needed.

## Resumo

A bartonelose é uma zoonose transmitida por vetores, com distribuição mundial, que infecta várias espécies de mamíferos. Apesar dos estudos conduzidos no Brasil, informações sobre *Bartonella* em cães são escassas. Portanto, foi realizado um estudo retrospectivo para investigar a exposição a *Bartonella* sp. em cães cães, utilizando-se o ensaio de imunofluorescência indireta (IFA). Trezentas e trinta e cinco amostras de soro de cães, previamente testadas para patógenos transmitidos pelos vetores, *Toxoplasma gondii* e *Neospora caninum* foram avaliadas quanto à presença de anticorpos IgG contra *Bartonella* sp. Todos os cães eram oriundos da região metropolitana de Ribeirão Preto, Nordeste do Estado de São Paulo. Vinte e oito amostras (8,3%) foram positivas para *Bartonella* sp. no ponto de corte de 64. Entre as 28 amostras positivas, 16 (57,1%) também foram positivas para *Ehrlichia canis*, 12 (42,8%) para *Babesia vogeli*, cinco (17,8%) para *T. gondii* e três (10,7%) para *L. infantum* e *N. caninum*. Os resultados demonstraram que os cães amostrados foram expostos a *Bartonella* sp. Como os animais eram de proprietários particulares, nossos achados demonstram que as pessoas também podem ter sido expostas a *Bartonella* sp. São necessários estudos para avaliar se a infecção por *B. canis* ou *E. canis* constitui fator de risco para infecção por *Bartonella* em cães.

## Introduction

*Bartonella* genus comprises vector-borne Gram-negative intracellular α-proteobacteria with worldwide distribution and recognized as emerging and re-emerging pathogens (Breitschwerdt, 2017). An increasing number of *Bartonella* species are recognized as zoonotic pathogens (Gutiérrez et al., 2015), including some species that has pets as primary reservoirs or accidental hosts (Chomel et al., 2006).

Domestic cats are the principal reservoirs of *Bartonella henselae*, the main agent of cat-scratch disease (CSD) (Chomel et al., 2006). Furthermore, several studies have highlighted the role of dogs as potential reservoirs of *B. henselae* (Alvaréz-Fernández et al., 2018; Mazurek et al., 2019). Unlike cat-related *Bartonella* species, no vector has been identified so far in the natural transmission cycle of *Bartonella* among dogs. However, based on distinct investigations, ticks and fleas have been proposed as potential vectors for *Bartonella* transmission among dogs (Cotté et al., 2008; Lashnits et al., 2022).

Dogs infected with *Bartonella* spp. can develop endocarditis, peliosis hepatis, granulomatous lymphadenits, weight loss, and epistaxis (Breitschwerdt & Kordick, 2000; Chomel et al., 2006). Beyond the impact on animal health, infection in domestic animals may result in a substantial reservoir of *Bartonella* in an urban environment that can serve as a source for human infection (Breitschwerdt & Kordick, 2000). Therefore, monitoring the exposure of these animals to zoonotic pathogens such as *B. henselae* is important.

Even with the questionable specificity of immunofluorescent antibody assay (IFA), this approach is frequently used for the diagnosis of bartonellosis in humans and animals (La Scola & Raoult, 1996; Neupane et al., 2018).

Despite the recent studies carried out in Brazil, data concerning bartonellosis in dogs from our territory are scarce. Previous works performed in Brazil showed serological prevalence ranging from 2% (4/197) to 24.7% (27/109) (Diniz et al., 2007; Fontalvo et al., 2017). Based on molecular methods, in addition to *B. henselae*, *Bartonella vinsonii* subsp. *berkhoffii* (Diniz et al., 2007) and *Bartonella clarridgeiae* (André et al., 2019) have already been reported in dogs from Brazil.

Given the limited number of *Bartonella* studies in dogs performed in Brazil, the purpose of this study was to investigate the exposure to *Bartonella* in dogs with suspected canine vector-borne diseases (CVBD), toxoplasmosis and neosporosis.

## Material and Methods

### Animals

This retrospective study used 335 dog serum samples from dogs with suspected vector-borne diseases, toxoplasmosis and neosporosis submitted to the Laboratory of Immunoparasitology of the Faculdade de Ciências Agrárias e Veterinárias (FCAV/UNESP). Available patient information includes only date of collection, site of origin of the animal and, in some cases, gender. Out of 335 dogs, gender was recorded for 294 animals (185 females and 109 males). Information related to dog breed, age, antibiotic treatment, arthropod control and outdoor activity was not available. Dog serum samples were excluded if a sample from the same animal was submitted within the prior four weeks, to exclude samples from convalescent animals. All dogs were sampled in the Metropolitan region of Ribeirão Preto, northeast of the State of São Paulo, and the samples were collected between April 2021 and April 2022. The dog serum samples were selected for convenience. Once sent to the laboratory, the samples were stored at -20 °C. During all serological analysis, the serum samples were subjected to a maximum 4 freeze-thaw cycle.

### Serological analysis

The serological detection of IgG antibodies to *Ehrlichia canis* (by ELISA - immunoabsorbent assay), *Leishmania infantum* (by IFA), *Babesia vogeli* (by ELISA), *Toxoplasma gondii* (by IFA) and *Neospora caninum* (by IFA) was carried out using the IMUNOTEST assays developed by IMUNODOT® Diagnostics, in accordance with the manufacturer’s recommendations.

### Detection of IgG antibodies to *Bartonella* sp. by IFA

In order to check for the presence of IgG antibodies to *Bartonella* sp. in dog serum samples, IFA was performed using the *B. henselae* ST9 antigen (Furquim et al., 2021). Previously frozen stocks of isolates were grown onto chocolate agar plates. Once colonies were plentiful, the bacterial cultures were passed into a confluent tissue culture flask containing DH82 cells (a canine monocytoid cell line). *Bartonella henselae* ST9 isolate used for antigen preparation in our study had not been passaged more than 3 times. The flasks were incubated for 4-5 days at 37 °C with 5% CO_2_ in a water-jacketed CO_2_ incubator (NuAire, Plymouth, Ma). After this period the supernatant was removed and the flasks were washed with balanced saline solution, calcium and magnesium free (BSS.CMF – pH 7.4) and trypsinized. The content was centrifuged at 3,000 x rpm for 10 minutes. The supernatant was discarded, and the cell were resuspended in PBS. Posteriorly, the cellular preparations were diluted to achieve a single layer of evenly-spaced cells. Formaldehyde (2%) was used for bacteria inactivation and to keep the cellular membranes intact. One hundred and twenty microliters of the cell cultures were spotted onto glass slides (12-well – 10 microliters per well). The slides were stored at -20 °C until the use.

Immunofluorescence reactions were performed as previously established (Henn et al., 2007). Briefly, dog serum samples were diluted (1:64) in PBS (pH 7.2) and 10 µL were added to individual wells of the slide containing the *B. henselae* antigen. After incubation of the slide in a humid chamber for 30 minutes at 37 °C, three washes were performed with PBS. After drying, the slides were incubated for 30 minutes at 37 °C with the anti-dog IgG conjugate (KPL) (1:32) coupled to fluorescein isothiocyanate and diluted with PBS and Evans Blue (10%). Subsequently, three washes were performed with PBS + 0.01% Tween 20. Finally, the slides were mounted with coverslips, with the addition of buffered glycerin, and analyzed using a UV microscope at 40X objective (Olympus, BX-FLA). Serum samples from cats were used as negative and positive controls (Furquim et al., 2021).

### Statistical analysis

The Pearson’s Chi-squared test was used to evaluate the association between gender and the number of dogs seropositive for *Bartonella* sp. using the SAS program (Statistical Analysis System version 9.2). The significance level was set at *p*<0.05

## Results and Discussion

Over 12 months, from April 2021 to April 2022, 335 serum samples from dogs were submitted to serological detection IgG antibodies to vector-borne pathogens (*E. canis, B. vogeli* and *L. infantum*), *T. gondii*, *N. caninum* and after that for *Bartonella* sp. Out of 335 serum samples, 28 (8.3%) were positive for *Bartonella* sp. All seroreactive dogs had low antibody titers (64).

Previous studies aiming to detect antibodies against *Bartonella* spp. in dogs from Brazil reported prevalence ranging from 2% (4/197) to 24.7% (27/109) (Diniz et al., 2007; Fontalvo et al., 2017). Since the sampling procedures in the different studies carried out in Brazil were distinct, as well as the IFA assay and antigens used, any comparison regarding the prevalence among the studies is purely speculative. In a retrospective study performed in the USA that analyzed 15,295 canine serum samples, Lashnits et al. (2018) reported that the region was a significant factor for seroreactivity against *Bartonella* spp. Based on logistic regression, dogs from the New England, Pacific, and West South-Central regions were more likely to be seropositive to *Bartonella* spp. than dogs from the South Atlantic region. However, the authors did not find seasonal trend in seroreactivity throughout the year (Lashnits et al., 2018).

In our study, a higher prevalence of IgG antibodies to *Bartonella* sp. was observed in females (10.3% - 19/185) than in male dogs (6.4% - 7/109), however this difference was not statistically significant (χ^2^ = 1.26, *p* = 0.261). Likewise, no statistically significant difference in *Bartonella* seroreactivity based upon gender was observed among dogs sampled in the USA and China (Lashnits et al., 2018; Song et al., 2020). On the other hand, Lashnits et al. (2018) demonstrated that male intact dogs had significantly higher seroreactivity than male neutered male dogs. In another study, the same group showed that dogs 1 to 5.5 years old were more likely to be *Bartonella* seroreactive than dogs <1-year-old (Lashnits et al., 2022).

Co-exposure to *Bartonella* and other pathogens in dogs, mainly vector-borne pathogens, has previously been reported (Breitschwerdt et al., 1998; Foley et al., 2007). These findings are consistent with those of the current study. Herein, out of 28 *Bartonella* seropositive samples, 16 (57.1%) were also seropositive for *E. canis* (whose absorbance values ranged from 0.432 to 1,470 [cut-off = 0.357]), 12 (42.8%) for *B. vogeli* (whose absorbance values ranged from 0.415 to 0.782 [cut-off = 0.330]), five (17.8%) for *T. gondii* (whose titers ranged from 40 to 640) and three (10.7%) for *L. infantum* (with titers of 40) and *N. caninum* (whose titers ranged from 40 to 320). Despite the fact that vector-associated transmission of *Bartonella* spp. in dogs is merely speculative (Angelakis et al., 2010; Mosbacher et al., 2011), the high level of co-exposure to *Bartonella, E. canis* and *B. vogeli*, the last two species known to be transmitted by *Rhipcephalus sanguineus* (Shaw et al., 2001; Bremer et al., 2005), continues to support ticks as possible vectors for *Bartonella* among dogs. In this way, given the high likelihood of CVBD co-exposure, screening for *Bartonella* could be considered in dogs infected with other CVBD.

Our retrospective study had some limitations based on the use of a convenience sampling. Since we only analyzed biological samples that were obtained from dogs that were evaluated at veterinary clinics instead of being collected randomly, the data of co-exposure should be interpreted with caution. Because healthy dogs may be less likely to have examined at veterinary clinics than ill dogs, and thus sick animals may be overrepresented in our study, the co-exposure observed herein may be overestimated.

Other limitation includes the use of only one *Bartonella* antigen (*B. henselae* ST9) to evaluate the exposure of dogs to *Bartonella*. Considering that dogs may be infected by at least 11 *Bartonella* species (Alvaréz-Fernández et al., 2018), the screening of the dog serum samples with other *Bartonella* antigens might have improved the number of seropositive dogs, getting closer to the “true” seroprevalence. Therefore, due to the possibility of dog infection with distinct *Bartonella* species and cross-reactivity between antibodies against *B. henselae* (the antigen used in this study) and other *Bartonella*, along with the low antibody titers herein observed, the IFA results provided by our study should be interpreted with caution.

The incidence of vector-borne pathogens has been increasing worldwide as a result of the environmental changes and alterations in demography and human behavior (Savić et al., 2014). The spreading of these pathogens of veterinary and human importance has become a critical threat in several parts of the world, including in Brazil. In this way, the constant monitoring of host/reservoir animals is a crucial point for the development of control measures that aim to reduce the risk of transmission of these agents among animals and humans, both in rural and urban areas.

Given that dogs serve as reservoirs or harbor zoonotic pathogens (Shaw et al., 2001), including *Bartonella* species (André et al., 2019), future surveillance studies focused on determining the incidence of *Bartonella* infection in dogs are crucial. To determine the actual prevalence of *Bartonella* in dogs, a combination of serological assays and an insect-based enrichment liquid-medium culturing approach followed by molecular techniques (qPCR and PCR) should be used. This approach was found to be beneficial and may improve the sensitivity of *Bartonella* species detection in dogs (Pérez et al., 2011).

## Conclusion

This retrospective study demonstrated that dogs from the Metropolitan region of Ribeirão Preto, southeaster Brazil, were exposed to *Bartonella* sp. Due to the fact that dogs sampled in the present study were from private owners, our findings suggest that these people may also be exposed to zoonotic pathogens.
